# Mechanisms of Control of *Mycobacterium tuberculosis* by NK Cells: Role of Glutathione

**DOI:** 10.3389/fimmu.2015.00508

**Published:** 2015-10-05

**Authors:** Michael Allen, Cedric Bailey, Ian Cahatol, Levi Dodge, Jay Yim, Christine Kassissa, Jennifer Luong, Sarah Kasko, Shalin Pandya, Vishwanath Venketaraman

**Affiliations:** ^1^College of Osteopathic Medicine of the Pacific, Western University of Health Sciences, Pomona, CA, USA; ^2^Department of Basic Medical Sciences, Western University of Health Sciences, Pomona, CA, USA

**Keywords:** natural killer cells, macrophages, *Mycobacterium tuberculosis*, HIV infections, AIDS-related opportunistic infections, glutathione, innate immunity

## Abstract

Tuberculosis (TB), caused by *Mycobacterium tuberculosis* (*M. tb*), continues to be one of the most prevalent infectious diseases in the world. There is an upward trend in occurrence due to emerging multidrug resistant strains and an increasingly larger proportion of immunocompromised patient populations as a result of the acquired immunodeficiency syndrome pandemic. The complex and often deadly combination of multidrug resistant *M. tb* (MDR-*M. tb*) along with human immunodeficiency virus (HIV) puts a significant number of people at high risk for pulmonary and extra-pulmonary TB without sufficient therapeutic options available. Natural killer (NK) cells and macrophages are major components of the body’s innate immune system, contributing significantly to the body’s ability to synergistically inhibit the growth of *M. tb* in immune compromised individuals lacking a sufficient T cell response. Direct mechanisms of control are largely through the secretory products perforin, granulysin, and granzymes, as well as multiple membrane-bound death receptors that facilitate target directed lysis. NK cells also have a role in indirectly stimulating an immune response through activation of macrophages and monocytes with multiple signaling pathways, including both reactive oxygen species and reactive nitrogen species. Glutathione (GSH) has been shown to play a part in inhibiting the growth of intracellular *M. tb* through bacteriostatic mechanisms. Enhancing cellular GSH through several cytokines and *N*-acetyl cysteine has been shown to increase these effects, at least in part, through their action on NK cells. Taken together, there is substantial evidence for a mechanistic correlation between NK cell activity and functionality in combating *M. tb* in HIV infection mediated through adequate GSH production and use.

## Epidemiology of Tuberculosis, Drug Resistance, and HIV Co-Infection

*Mycobacterium tuberculosis* (*M. tb*), the causative agent for tuberculosis (TB), spreads from person to person through airborne transmission. The spread of MDR-*M. tb* and the increased number of immunocompromised individuals due to the acquired immunodeficiency syndrome (AIDS) pandemic have led to an increasing incidence of *M. tb* infection (1–4). TB remains an important public health problem with 9 million new cases and 1.5 million deaths in 2013 (5). Of these deaths, 0.36 million people were also co-infected with human immunodeficiency virus (HIV) (6).

Drug resistance poses a significant challenge to TB control and treatment. MDR-*M. tb* and extensively drug-resistant TB (XDR-*M. tb*) require longer treatment time, and more expensive second-line anti-TB drugs with additional side effects. Additionally, cases of “totally drug-resistant TB” (TDR-TB) have been reported globally (7–9).

Patients with AIDS due to HIV infection are susceptible to opportunistic pathogens, with TB being the most common and perhaps the leading cause of death in these patients (1, 2, 5). There is a dangerous relationship between TB and HIV co-infection, with HIV increasing the likelihood of TB infection, and TB worsening the clinical course of HIV infection (2). In immunocompromised individuals with impaired adaptive immunity, natural killer (NK) cells could serve as the major immune cells contributing to innate defense against bacterial infection (4, 10).

## *M. tb* Infection and Host Immune Responses in the Lungs

Initial *M. tb* infection occurs in the lungs, where invading *Mycobacterium* are phagocytosed by alveolar macrophages. Binding of toll-like receptors (TLRs) triggers the release of inflammatory mediators of the innate immune response. Neutrophils, NK cells, and eventually T-cells respond to these signals and are thought to contribute to early defenses against *M. tb* infection (11). These early responses frequently prove unsuccessful in combating the initial infection. In such cases, the infection spreads via macrophages and dendritic cells (DCs) to the draining lymph nodes, through the bloodstream to various organs. However, the initial infection only progresses to overt disease in a small number of cases. In most immune competent patients, granulomas form to contain the infecting *Mycobacterium*, driven by proliferation of CD4 and CD8 T-cell lineages (11). Within the granulomas *M. tb* cells are contained without being completely eradicated, leading to a subclinical persistent infection, termed latent TB infection (LTBI). Immunodeficiency due to HIV infection represents the greatest recognized threat to successful containment of LTBI (1). Therefore, both innate and adaptive immune responses are extremely important for in combating *M. tb* infection (4, 12, 13). Activated alveolar macrophages provide the first-line defense against *M. tb* infection by phagocytosing the bacteria and mounting appropriate effector mechanisms within the intracellular environment, thereby limiting the growth of the pathogen and preventing overt disease (10, 13). Although NK cells are not phagocytic, these cells can still play a pivotal role in innate defense against *M. tb* infection by various mechanisms described in this review (2, 10, 14–27).

## Natural Killer Cells and Cytotoxic Mechanisms

Natural killer cells are granular lymphocytes that express CD16, an Fc receptor, and CD56, a neural cell adhesion molecule (NCAM) (28). NK cells carry out non-MHC-restricted cytotoxicity against pathogens along with host cells that have lowered or absent expression of MHC class I molecules (29, 30). The cytolytic functions of NK cells are regulated by the engagement of both activating and inhibitory cell-surface receptors (30). MHC class I molecules are expressed by nucleated cells and engage inhibitory NK cell receptors, such as killer cell Ig-like receptors (KIRs), leukocyte Ig-like receptor (LIR), and CD94/NKG2 receptors, to prevent cytolysis (31, 32). CD94 is a C-type lectin protein, which can not only dimerize with NKG2 family proteins to create an inhibitory receptor but can also dimerize with NKG2C, -E, and -H and function as an activating receptor. Most of the CD94/NKG2 receptors are heterodimers, which bind to the non-classical class I molecule HLA-E (31, 32). NK cells bear a variety of activating receptors, including NKp30, NKp44, and NKp46, the surface expression of which is associated with increased lysis of target cells (6, 33, 34).

Cytokines, such as IL-2 and IL-12, have both been shown to increase and activate NK cell activity. IL-2 enhances the lytic activity of NK cells, commonly referred to as lymphocyte activated killer (LAK) activity (4). The cytolytic activity of NK cells has also been demonstrated with IL-2 treatment on macrophages infected with *Legionella*, *Shigella*, and *Rickettsia* (35). IL-12 is a powerful NK cell stimulatory factor secreted by macrophages, neutrophils, and DCs after exposure to pathogens or cytokines(22). IL-12 signaling increases NK cell function and promotes synthesis and secretion of interferon gamma (IFN-γ). NK cells activated by IL-12 have been shown to inhibit the growth of *M. tb* and *Mycobacterium avium* (19, 22).

Two suggested mechanisms help explain the involvement of NK cells in the defense against microbes: direct and indirect. NK cells bind to microorganisms and provide direct cytotoxicity via secreted bactericidal molecules (19). The expression of surface ligands on NK cells provides the capability of killing pathogens and target cells by inducing apoptosis (36–40). Through secreted cytokines, such as IFN-γ and tumor necrosis factor-alpha (TNF-α), NK cells can activate macrophages and other members of the immune system, resulting in the indirect killing of pathogens.

## Direct Mechanisms of NK Cell-Mediated Control of Infection

**Figure 1:**
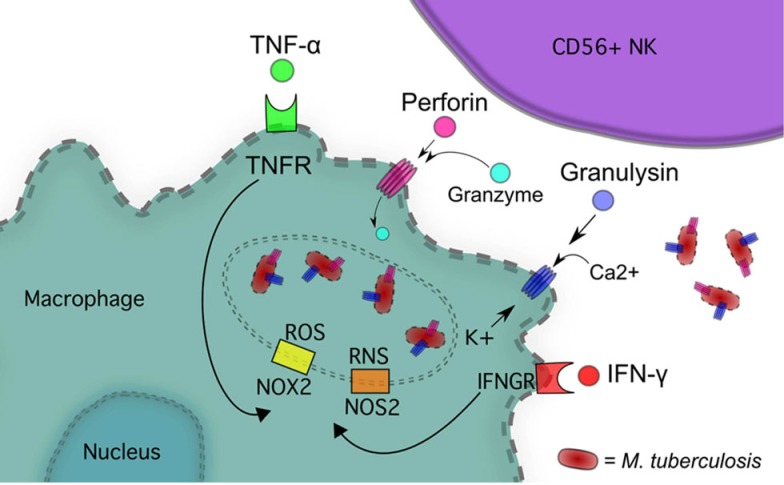
**NK cells inhibit *M. tb* growth via IFN-*γ*, TNF-*α*, Perforin and Granulysin, and Granzyme**.

Natural killer cells have several direct mechanisms of cytotoxicity, including cytoplasmic granules containing perforin, granulysin, and granzymes, as well as several death receptors that can initiate apoptosis (Figures 1 and 4). Perforin belongs to the membrane-attack-complex protein family, and inserts into target cellular membranes to function as a pore similar to the c5–9 membrane attack complex of the complement system (41). Perforin pores are used to facilitate transport of granulysin and granzyme into the target cell cytoplasm. Additionally, perforin may serve other cytolytic functions in the target cell after the transport of cytolytic molecules (42). Granzymes are a family of serine proteases with many members, the major constituent in NK cells being Granzyme B. This enzyme can initiate apoptosis of the target cell through direct activation of Caspases 3 and 7 or through proteolysis of the protein Bid. Cleavage of Bid allows the active fragment to move to the mitochondrial membrane and form a pore complex with Bax and Bak, promoting the exit of cytochrome *c* into the cytosol, thereby initiating the formation of the caspase-activating complex (43). Granzyme B can also mediate nuclear destruction in the presence of perforin, and other granzymes may be able to mediate caspase-independent cell death pathways (42). Although granzymes can be taken into target cells without perforin, full effects of these enzymes are not observed unless perforin is present (42).

Granulysin shares homology with a family of proteins called saposins, acid sphingomyelinase, and several Amoebapore proteins from *E. histolytica*. These proteins all have the ability to interact with lipids. Two isoforms of granulysin exist in NK cells, a 15-kDa form that is constitutively secreted and a 9-kDa form found in cytolytic granules (44). Granulysin is broadly antimicrobial and tumoricidal. Able to kill extracellular *M. tb* on its own, granulysin is not effective against intracellular *M. tb* without the presence of perforin in which case it is highly effective at disrupting lipid metabolism and oxidative phosphorylation (44, 45). Damage to lipid membranes leads to release of cytochrome *c* into the cytosol initiating apoptosis along with generating reactive oxygen species (ROS) (46).

Fas (APO-1/CD95) is a TNF receptor family trans-membrane death receptor responsible for cell lysis, whose ligand (FasL) is expressed in NK cells (37, 47). The Fas receptor can be found on most cell types in the body, and is of particular interest in the context of macrophage expression. Upon FasL–Fas ligation a death-inducing signaling complex (DISC) forms, composed of multiple proteins, including Fas, Fas-associated death domain (FADD), and caspase-8. Activation of caspase-8 by DISC initiates the extra-mitochondrial apoptotic pathway (47). Macrophages infected with *M. tb* undergo NK-mediated apoptosis through this Fas pathway, to limit viability of *M. tb* (48).

Natural killer cells express CD40L, the ligand for CD40 that is expressed on antigen presenting cells and macrophages. (38, 39). Engagement of CD40 is central to the initiation of the immune response. After ligation, CD40 leads to up-regulation of co-stimulatory molecules CD80 and CD86 on the cell surface of macrophages as well as generation of nitric oxide (NO) when accompanied by IFN-γ (49). HIV infection demonstrates a significant relationship between CD40 expression and immunity to opportunistic pathogens. *Candida albicans*, cytomegalovirus, and *Toxoplasma gondii* infection concomitant with HIV results in cells with impaired CD40 expression and function (40). With exposure to *T. gondii*, cells from HIV-infected patients have decreased production of IL-12 and IFN-γ that can be partially restored with recombinant CD40. This effect on CD40 most likely contributes to the increased susceptibility of HIV-infected patients to opportunistic pathogens, by diminishing the activity of cell-mediated immune responses (40).

Natural killer cell production of IFN-γ and TNF-α increases the cytolytic ability of the NK cell by up-regulating ICAM-1 adhesion molecules in target cells through activation of the NF-κB pathway (50). ICAM-1 expression allows for greater target cell adhesion to NK cells, increasing cytolysis.

## Indirect Mechanisms of NK Cells and IFN-γ-Induced Effector Mechanisms in Macrophages

Interferon gamma released by NK cells can trigger numerous intracellular effector mechanisms within macrophages, such as activation of NADPH-oxidase type 1 and 2 (NOX1, 2) as well as NO synthase type 2 (NOS2), leading to formation of ROS and reactive nitrogen species (RNS), respectively (51, 52) (Figure 1). NOS2 expression in humans has been controversial but recent studies have demonstrated the expression of this isoform after immune cell activation (53–55). IFN-γ also up-regulates the expression of IgG Fc receptor FcγRI in monocytes to increase opsonization dependent phagocytosis (51).

NOX2 is recruited to phagosomes by IFN-γ stimulation where it catalyzes the production of superoxide $\left({\text{O}}_{2}^{-}\right)$ from O_2_ and NADPH (56, 57). NOS2 catalyzes the conversion of l-arginine and O_2_ into NO and citrulline (58). TNF-α released by NK cells also contributes to macrophage mitochondrial ROS formation through TNF receptor 1 (TNFR1) complex association with NOX1, induction of NOX2, and through a receptor-interacting serine-threonine kinases 1 and 3 (RIP1-RIP3)-dependent pathway, which can lead to programed necrosis (59–61).

Superoxide can spontaneously generate hydrogen peroxide and hydroxyl radicals (56, 57). ROS and RNS can react further with each other to generate NO_2_ and peroxynitrite (OONO^−^) (56, 62, 63). These various reactive species contribute antimicrobial oxidative destruction to membrane lipids and proteins, DNA, and enzymes (56).

## Nitrosoglutathione (GSNO) is More Potent than NO Alone

Although an unquestionably powerful antimicrobial agent, the limitations of NO are clear. A short half-life limits its potent antimicrobial properties while protecting our cells from widespread, uncontrolled destruction. As it turns out, human cells have evolved a particularly useful method of keeping NO in reserve for immediate, long-lasting effects with a slow-release. In a process known as S-nitrosylation, NO reacts with the tripeptide antioxidant glutathione (GSH) to form *S*-nitrosoGSH (GSNO). GSNO acts as a donor of NO, releasing it to the cell as needed.

In the form of GSNO, NO can better spread throughout the system, being readily transported to other parts of the body. This mechanism proves particularly powerful during an infection, where macrophage activation utilizes massive quantities of NO. The superior functions of GSNO are well evidenced, with studies indicating a cidal effect of GSNO on *M. tb* by means of its “as-needed” release of NO. GSNO has also been found to protect against oxidative stress caused by peroxynitrite in brain, as well as oxidative stress-induced apoptosis through induction of C-GMP-mediated synthesis of thioredoxin. Both the destructive and protective functions of GSNO represent a more potent and stable mechanism through which the cell can utilize the powerful effects of NO in the immune system. GSNO has therefore been shown to play a major role in macrophage function in controlling *M. tb* growth (63, 64).

## Natural Killer Cells in *M. tb* and HIV Infection

Natural killer cells isolated from healthy donors have been shown to lyse infected monocytes and reduce the rate of intracellular *M. tb* growth (22, 25). Depletion of NK cells in a murine model enhances the growth of *M. avium* (21). However, one study has revealed that though NK cells are expanded in C57BL/6 mice in response to *M. tb* infection, there is no increase in bacterial load when NK cells were depleted, suggesting that there is redundancy in the cellular response to mycobacterial infection (15). Interestingly, in T-cell deficient mice infected with *M. tb*, NK cells are required for mycobacterial resistance, suggesting an important role for NK cells in combating *M. tb* in immunocompromised individuals (17).

Growth inhibition of mycobacteria is, in part, mediated by soluble factors, including IFN-γ and TNF-α produced by NK cells, and through monocyte apoptosis induced by NK cell contact (23, 24). NK cells are capable of destroying *M. tb* via the cytolytic proteins granulysin and perforin, in a contact-dependent manner without the help of accessory cells, such as monocytes (27) (Figure 1).

In addition to early innate immune functions, NK cells are found in mature granulomatous lesions in the lungs of *M. tb* infected patients (18). Direct interactions between NK cells and *M. tb* that emerge from granulomas after cell lysis can initiate antimicrobial mechanisms, such as discharge of granulysin, with static or cidal effects on the growth of *M. tb* (16, 18, 27). In the presence of IL-2 and/or IL-12, NK cells express NKp44 leading to direct interactions with the corresponding ligand on the cell-surface of *M. tb* infected macrophages, triggering IFN-γ production and increased macrophage activation (16, 18). Exposure of NK cells to *M. tb* up-regulates the expression of NKG2D as well as NKp30, NKp44, and NKp46, which then increase the expression of granulysin and perforin through the MAP kinase signaling pathway (27). After *M. tb* challenge, NK cells up-regulate nanotube-like structures that tether mycobacteria, and redistribute perforin vesicles toward these tethered regions (27).

FasL-induced apoptosis of *M. tb* infected macrophages leads to greatly reduced viability of intracellular *M. tb* (48) (Figure 4). HIV is able to de-regulate FasL and delay Fas-induced apoptosis of infected peripheral blood lymphocytes leading to prolonged infection (36). This may contribute to increased viability of *M. tb* in HIV-infected patients. NK cell cytolytic function has been shown to be impaired in viremic HIV-1 infected subjects, potentially contributing to the escape of infected CD4^+^ T cells from NK cytotoxicity (65). This impairment is associated with an increased surface expression of inhibitory receptors, such as LILRB1 and NKG2A (31, 65, 66). Investigations also suggest surface expression of natural cytotoxicity receptors NKp46 may be elevated or diminished, whereas NKp30 and NKp44 may be unchanged or diminished in HIV-1 infected patients (66–68). Stimulation of HIV-1 infected cultures with IL-15 and IL-12 has shown replenishment of NK cell populations, which are selectively lost during HIV infection (68).

HIV-1 infection has been shown to modulate the subsets of IFN-γ producing NK cells in response to bacteria. A lower overall frequency of IFN-γ producing NK cells is seen from HIV-1 infected donors responding to *E. coli* and *S. typhimurium*, than NK cells from uninfected donors (69). The decrease in bacteria reactive, IFN-γ producing NK cells in HIV-1 infected subjects contributes to the compromised innate immune response to opportunistic infections.

## The Role of Glutathione in the NK Immune Response

**Figure 2:**
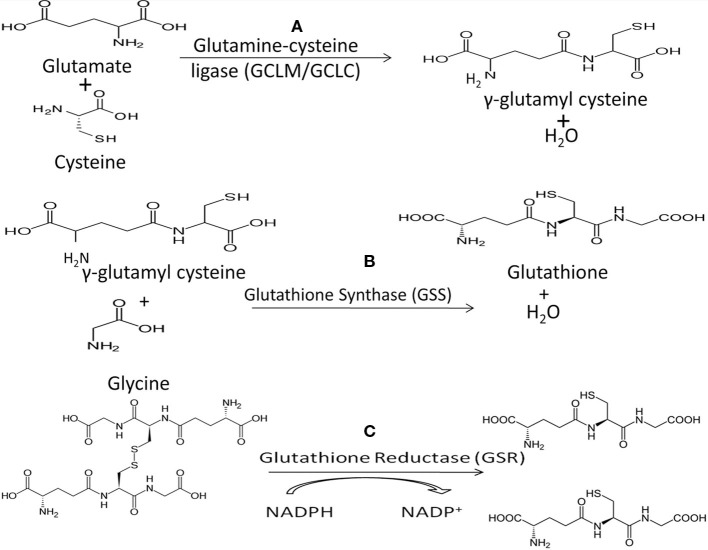
**(A)** The first step in *de novo* GSH biosynthesis is the rate-limiting step. Glutamate and cysteine are covalently linked by the heterodimeric enzyme glutamylcysteine ligase (GCL) to form γ-glutamylcysteine. Notably, cysteine’s sulfhydryl bond is the source of GSH’s antioxidant capacity. **(B)** GSH synthase (GSS) catalyzes the second step in GSH biosynthesis by linking glycine and γ-glutamylcysteine to form GSH which functions as the substrate for GPx in reduction of H_2_O_2_. **(C)** Oxidized GSH, in the form of GSSG, can be converted to free GSH by glutathione reductase (GSR), utilizing NADPH as a cofactor.

Low levels of intracellular GSH dramatically decrease NK cell cytotoxic functions (4, 26, 70). GSH is the main non-protein thiol responsible for cellular homeostasis and oxidative balance (71). GSH exists in oxidized (GSSG) and reduced/free form (*r*GSH), with *r*GSH exhibiting antioxidant activity. Glutathione peroxidase (GPx) detoxifies hydrogen peroxide, a potent source of ROS within the cell, through a redox reaction with GSH (71, 72). *r*GSH can be reclaimed from GSSG by the activity of glutathione reductase (GSR) (Figure 2). Treatment with *N*-acetyl cysteine (NAC) results in increased intracellular GSH and treatment with buthionine sulfoximine (BSO) decreases GSH. Although the uptake of cysteine is considered as the rate-limiting step for the synthesis of GSH, the most efficient way to increase the levels of cysteine in cells is to supplement with NAC, which is easily taken up by the cells and is non-toxic (72). BSO specifically inhibits the activity of glutamate–cysteine ligase, the enzyme that catalyzes the first reaction in the synthesis of GSH thereby inhibiting its synthesis (Figure 2).

Notably, mycobacteria do not synthesize GSH and instead produce mycothiol to serve the same function. The exogenous nature of GSH to mycobacteria may contribute to observations that the virulent laboratory *M. tb* strain H37Rv is sensitive to GSH at physiologic concentrations *in vitro*, supporting the bacteriostatic nature of GSH (63, 70). Functionally the bacteriostatic activity of GSH likely stems from the redox imbalances of the bacterium’s metabolic homeostasis, which is normally regulated by the activity of mycothiol. Analysis of GSH’s inhibition of intracellular H37Rv within human monocyte-derived macrophages (HMDM) demonstrates the relationship between increased GSH and control of intracellular *M. tb* growth (10, 12, 13, 70, 73). Another proposed mechanism for the anti-mycobacterial nature of GSH is the significant structural similarities between GSH and penicillin antibiotics (74, 75). The above findings have led to the conceptual framework for the inhibition of intracellular H37Rv growth in HMDM by the direct anti-mycobacterial action of GSH (10, 12, 13, 70, 73).

Given this role of GSH it was further postulated that treatment of whole blood cultures with NAC would result in greater inhibition of H37Rv growth through both direct anti-mycobacterial effects and enhancement of NK cell activation. Whole blood cultures and blood cultures depleted of CD56^+^ NK were each treated with NAC and subsequently infected with processed H37Rv (4). Growth inhibition occurred in the presence of NK cells and NAC, whereas growth continued at different rates in all other cell cultures with NK cells or NAC alone.

The effects of GSH in enhancing the cytolytic activity of NK cells are demonstrated through cytotoxicity assays. NK cell cytolytic activity increases in the presence of NAC and further increases with the addition of cytokines. Treatment with NAC^+^IL-2^+^IL-12 exhibits the greatest cytolytic activity, compared with any individual component alone. It can therefore be concluded that GSH alone and with cytokines is an important component affecting NK cell cytotoxicity (4).

Furthermore, the effects of NAC^+^ cytokines are important in NK cell control of *M. tb* growth. Monocytes cultured in an environment without NK cells display a fourfold increase in intracellular growth of H37Rv, whereas monocytes co-cultured with NK cells display only a threefold increase of H37Rv (10). Treatment of NK cells with NAC results in additional growth inhibition of H37Rv (only a twofold increase in the growth compared to negative control). Treatment with IL-2^+^IL-12^+^NAC remarkably leads to growth stasis of H37Rv, whereas IL-2^+^IL-12 alone does not lead to such stasis, suggesting that NAC has a marked effect. Further reinforcing the link between GSH and NK cell function, NK cells grown with BSO which functionally blocks the GSH pathway, result in a maximal fivefold increase in H37Rv intracellular growth (10).

**Figure 3:**
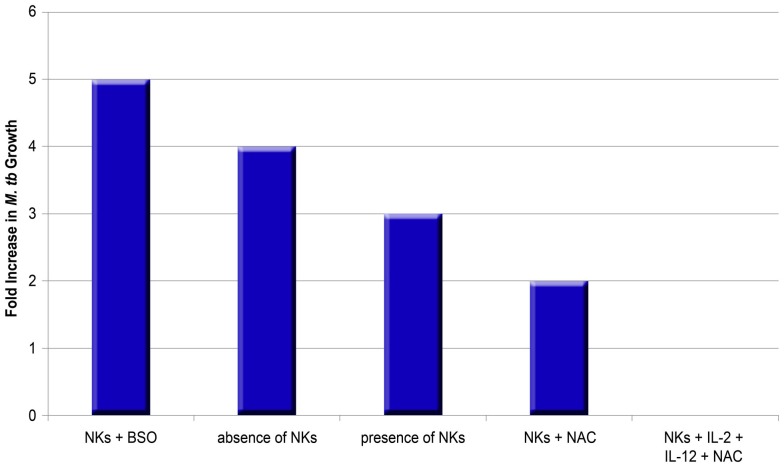
**Impact of NK cells on the growth of *M. tb* in human macrophages in the presence or absence of thiols and cytokines**. The graph summarizes data from different studies (10, 13). BSO inhibits the synthesis of GSH, whereas NAC enhances the availability of rGSH.

**Figure 4:**
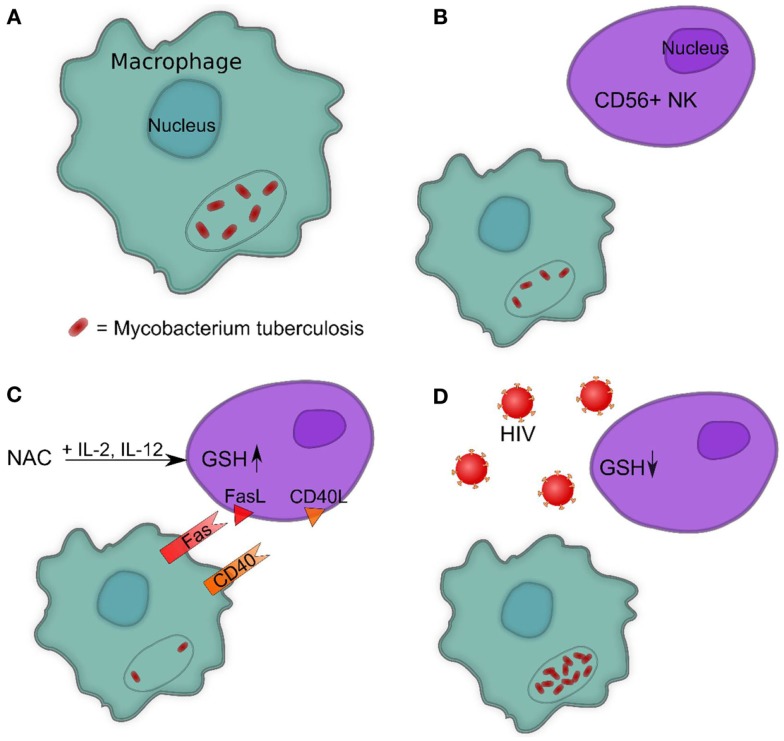
**(A)** Intracellular survival of *M. tb* inside human macrophages. **(B)** Presence of NK cells reduces the intracellular survival of *M. tb* inside human macrophages. **(C)** Increasing GSH in NK cells reduces the intracellular survival of *M. tb* inside human macrophages by promoting interactions between Fas–FasL and CD40–CD40L. **(D)** HIV infection decreases the levels of GSH in NK cells leading to enhanced survival of *M. tb* inside human macrophages.

The above findings indicate that GSH controls *M. tb* infection by its direct anti-mycobacterial effects as well as by activating NK cells in conjunction with IL-2^+^IL-12 (Figure 3) (10, 13). Notably, NK cell co-incubation with NAC and NAC^+^IL-2^+^IL-12 led to a measured increase in CD40L and FasL (10). GSH, along with IL-2^+^IL-12, clearly plays a role enhancing the ability of NK cells to express cytotoxic ligands and, as such, serve as markers for NK cell activation and regulate NK cell induction of apoptosis in targeted cells (Figure 4C).

FasL and CD40L have an observable role in monocytes’ control of *M. tb* infection, demonstrated by studies that leveraged neutralizing antibodies to block their functional role. Interestingly, blocking CD40L results in abrogation of the static effect of IL-2^+^IL-12^+^NAC on intracellular growth patterns of H37Rv, resulting in a threefold increase of intracellular H37Rv growth (10). Neutralization of FasL results in a fivefold increase in the intracellular growth of H37Rv, whereas monocytes co-cultured with untreated NK cells demonstrate a fourfold increase of intracellular H37Rv. NK cells rely on proper FasL and CD40L functioning and monocytes rely on Fas and CD40 functioning in order to effectively inhibit the growth of intracellular *M. tb*. Taken together, GSH has a significant and instrumental role in augmenting and enhancing NK cell functions, enhancing the ability of NK cells to limit intracellular infections inside monocytes (Figures 4B,C) (10).

## GSH Levels in NK Cells from Healthy and HIV-Infected Subjects

Human immunodeficiency virus-positive individuals tend to have much lower NK cell GSH concentrations in comparison to healthy subjects. (10). Although HIV infects CD4 T cells and monocytes, a significant decrease in the intracellular levels of GSH in NK cells has been observed. This is due to elevated levels of circulating TNF-α and ROI induced by HIV infection, which require the anti-oxidative protection of GSH (12, 73). Decreased GSH levels in NK cells from individuals with HIV infection is accompanied by increased multiplication of *M. tb* inside monocytes (Figure 4D).

A marked decrease in levels of GSH and rGSH was found in isolated macrophages in individuals with HIV infection compared to healthy subjects (12, 73). Even more interesting is the ratio of rGSH to GSSG. In the macrophages of HIV-infected individuals, total GSH was approximately 30% *r*GSH and 70% GSSG. However, the total GSH in macrophages from healthy subjects was approximately 60% *r*GSH and 40% GSSG (12, 73). Furthermore, the concentration of GSH in T-cells of HIV-positive individuals was much lower versus T-cells from healthy individuals (13). Diminished concentrations of GSH in macrophages, NK, and T cells derived from individuals with HIV infection was directly correlated to diminished control of intracellular *M. tb* infection (10, 12, 13, 73).

**Figure 5:**
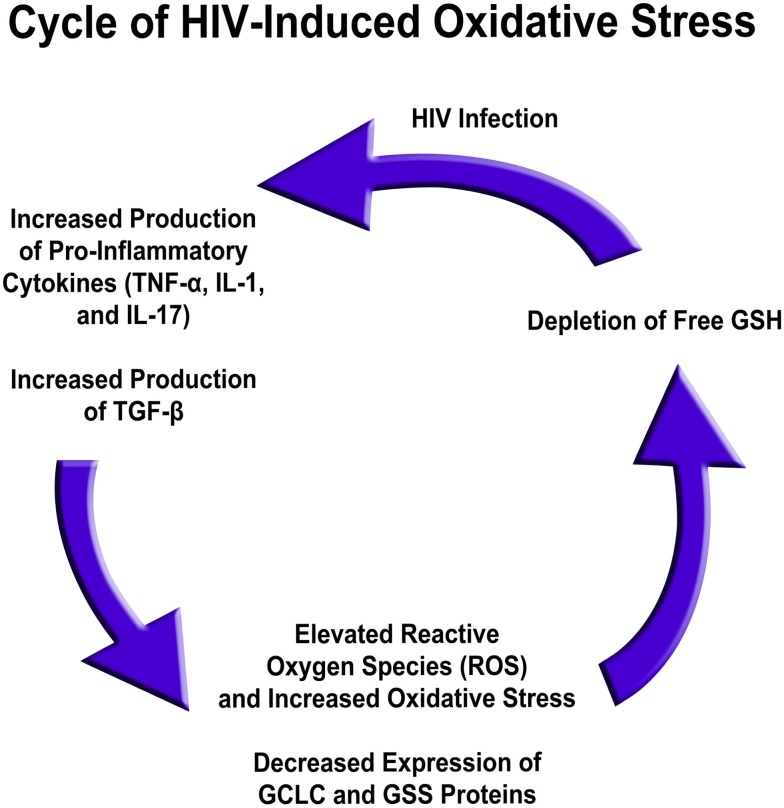
**HIV infection induced oxidative stress and redox imbalance in leading to enhanced susceptibility to *M. tb* infection**.

Several factors are responsible for low GSH in HIV infection. The levels of free radicals and pro-inflammatory cytokines, such as IL-1, IL-17, and TNF-α, are elevated in the plasma of HIV-positive individuals versus healthy individuals, whereas gene expression for enzymes responsible for *de novo* GSH synthesis are decreased in HIV-infected individuals’ macrophages (9). Based on these results, the excess production of pro-inflammatory cytokines in HIV-positive subjects likely leads to a rise in free radical levels (10, 12, 13, 73). This, in conjunction with diminished GSH synthesis enzyme expression, leads to a net effect of rGSH depletion, possibly contributing to diminished immunity in HIV-positive individuals (Figure 5) (76).

## Summary

Natural killer cells help control *M. tb* growth through multiple mechanisms and play a particularly important role in immune compromised individuals that lack the ability to mount a sufficient T cell response. These mechanisms include release of cytokines, such as IFN-γ and TNF-α, production of cytolytic proteins, such as perforin and granulysin, and direct interactions between CD40/CD40L and Fas/FasL on the mycobacteria surface (10, 23, 27, 37). Recent studies indicate that NK cells can be activated by IL-2, IL-12, and GSH to control *M. tb* infections (10, 13). GSH also functions to limit the intracellular growth of *M. tb* in human and murine peripheral blood-derived monocytes, likely through direct anti-mycobacterial activity, functioning as an effector in innate defense and by activating immune cells (4, 10, 12, 13, 70, 73). NK cell function and intracellular GSH levels are significantly compromised in HIV-infected individuals, which is critical given the increased susceptibility of HIV-infected individuals to *M. tb* infection, particularly disseminated disease (4).

Upward trends in the global incidence of TB are largely the result of the emergence of MDR and XDR *M. tb* strains and an increasing number of immunocompromised individuals due to the AIDS pandemic. These phenomena raise the likelihood that drug-susceptible TB may eventually be replaced by largely drug-resistant TB, thereby severely restricting treatment modalities. As there have been no new drug therapies targeted at TB during the past 40 years, immuno-adjunctive therapy holds a promising role in potentially expanding treatment options and outcomes for refractory *M. tb* infections. By exploring the role of NK cells in HIV and TB infection, it is clear that immunotherapies enhancing the function of NK cells will play a role in treatment in conjunction with anti-mycobacterial drugs. We anticipate that research in the coming years will find a likely role for GSH as a highly promising immune-adjunctive component of treatment protocols for MDR-TB, particularly in immunocompromised individuals suffering from HIV.

## Conflict of Interest Statement

The authors declare that the research was conducted in the absence of any commercial or financial relationships that could be construed as a potential conflict of interest.
